# Impact of a CBT psychotherapy group on post-operative bariatric patients

**DOI:** 10.1186/s40064-015-1558-6

**Published:** 2015-12-09

**Authors:** Julie Beaulac, Daniella Sandre

**Affiliations:** Department of Psychology, The Ottawa Hospital, Ottawa, ON Canada; Clinical Epidemiology, Ottawa Hospital Research Institute, Ottawa, ON Canada

**Keywords:** Bariatric surgery, Psychotherapy, Outcomes, Coping, Adjustment, Emotional eating

## Abstract

Psychological difficulties for patients seeking bariatric surgery are greater and in the post-operative phase, a significant minority go on to experience significant psychosocial difficulties, increasing their risk of poorer post-operative adjustment and associated weight regain. 17 post-operative patients participated in an eight-week cognitive behavioral therapy (CBT) based psychotherapy group at the Ottawa Hospital. A pre-post design with a 3-month follow-up investigated the impact of the group on emotional eating, general as well as obesity-specific adjustment, psychological distress, and attachment. There were significant and meaningful improvements in patients’ level of psychological distress, perceived difficulties in their lives, and weight-related adjustment that were maintained at a 3-month follow-up period. Although statistical change was not significant, there were also meaningful improvements in emotional overeating and relationship anxiety and avoidance. The intervention also appeared to be acceptable to patients in that attendance and satisfaction were good. Findings suggest that a short-term CBT psychotherapy group led to significant and meaningful benefits in psychological wellbeing for post-surgical bariatric patients.

## Background

The Weight Management Clinic (WMC) of The Ottawa Hospital offers a range of treatments from lifestyle interventions to bariatric surgery. For bariatric surgery, patients are followed post-operatively for 5 years to monitor health and provide support, comprehensive care is viewed as integral to ensuring success with weight loss and maintenance (Pataky et al. 2011).

The current evidence on psychosocial predictors of post-operative success is inconclusive. What we do know is that patients who present for bariatric surgery have a higher than average rate of psychological problems as compared to the general population or as compared to individuals with obesity who do not present for bariatric surgery (Lier et al. 2011). Many patients experience improvements in psychological and interpersonal functioning post-surgery (Van Hout and Van Heck 2009) although, when surgical outcomes are not met, behavioural and psychological factors are common key contributors (Sarwer et al. 2008). Specifically, as many as 20 % of patients report suboptimal weight loss or weight regain at some point in the post-operative phase (Marcus et al. 2009). Some researchers have proposed that adjustment to post-operative status as well as various psychological factors are likely to be key contributors in these cases (Wimmelman et al. 2014). In particular, patients who eat in response to negative emotions are reported to experience poorer post-operative weight loss (Gentry et al. 1984). A significant minority of patients receiving bariatric surgery, go on to experience increased rates of psychological problems such as depression, anxiety, suicidal ideation and suicide, increased relationship problems, and problematic eating behaviours (Tindle et al. 2010).

Psychotherapy, and in particular, group psychotherapy, has been demonstrated to be an effective treatment for numerous psychological difficulties and the management of other conditions (Cahill et al. 2010; Hunsley 2002). Evidence on psychological interventions specific to the bariatric population post-operatively is in its infancy; however, studies have demonstrated that participation in group-based psychotherapy has a positive impact on weight loss outcomes among post-operative bariatric patients (Beck et al. 2012; Saunders 2004). Ashton and colleagues explored the use of a four-session cognitive behavioral therapy (CBT) based psychotherapy group to address binge eating behaviors among bariatric surgery candidates and found that the intervention was successful in reducing the frequency of these episodes (2009). The combination of CBT and mindfulness-based interventions has also been found useful in reducing binge eating and emotional eating behavior as well as in reducing eating-related concerns, improving affect regulation, and reducing self-reported depressive symptoms (Leahey et al. 2008). With this rationale in mind, a psychotherapy group was developed and implemented at the WMC for post-operative bariatric patients. Its aim was to assist patients in developing coping strategies for managing day-to-day stressors, interpersonal conflicts, and difficulties adjusting to post-operative status; to assist patients in increasing awareness of behavioural, cognitive, and physiological cues associated with negative affective states; to support patient attempts to maintain healthy behaviour change; and, to develop relapse prevention skills. Ultimately, the psychotherapy group aimed to improve post-surgical outcomes by addressing psychosocial issues that could contribute to poor post-operative adjustment and associated weight re-gain. This pilot study investigated the impact of a psychotherapy group on emotional eating, general and obesity-specific adjustment, psychological distress, and attachment.

## Methods

The interventions studied were two consecutive 8-week closed psychotherapy groups held at the WMC during the months of March–April 2013, and again in October–December 2013. The groups were facilitated by one of the clinic psychologists and a psychology resident and both followed the same protocol. The content of the group was loosely based on research studies which explored the benefits of cognitive behavioral interventions on problematic eating patterns among bariatric surgery patients (Ashton et al. 2009; Leahey et al. 2008) in addition to individually-focused CBT and psycho educational interventions aimed at preparing patients for surgery more broadly (Apple et al. 2006). Each week focused on a specific theme (e.g., stress and coping, emotional eating, changing negative thinking, body image, self-esteem, emotional eating). Session 1 involved an introduction to the CBT model of overeating as well as education on the stress response system. Session 2 explored the topic of coping, including action-oriented, emotion-focused, and avoidant coping styles. Relaxation techniques were introduced and practiced in vivo as an example of action-oriented coping. Session 3 explored the relationship between emotions and food. Education was provided on the purpose of emotions, learned responses for emotion regulation, as well as the neurobiological impact of food as it pertains to affect regulation. Session 4 reviewed specific strategies aimed at reducing emotional eating behavior, with a focus on problem solving and self-care. In this session, education was also provided on various forms of hunger (i.e., physical hunger, emotional hunger, and sensory hunger) and the importance of self-monitoring. Session 5 introduced the topic of negative self-talk and explored strategies for challenging and reframing cognitive distortions. Session 6 focused on body image and the role of negative self-talk in influencing body image dissatisfaction. Session 7 explored the topic of relationships and the roles of anger, assertiveness, and aggression as they pertain to communication styles. Strategies for improving communication were introduced. Session 8 focused on the concepts of relapse prevention, including identification of high risk situations, problem solving, and the role of self-efficacy and outcome expectancies.

Each session’s theme was introduced and topic-specific education provided in a semi-structured format. Discussion was encouraged and patients were invited to share their experiences with each topic area. Each session introduced the development of skills for addressing difficulties associated with the theme. “Homework” was assigned and members were encouraged to complete these assignments in order to practice the skills. A “check-in” and “check-out” was conducted at each session to provide an opportunity for sharing of reflections on material or difficulties between sessions.

Patients were referred by their WMC health care provider or self-referred. Patients who were more than 6 weeks post-surgery and experiencing psychosocial issues (e.g., relational issues, depressed mood) were included. Patients with unstable medical conditions, disruptive interpersonal styles, or who required specialized treatment (e.g., psychosis, active suicidal ideation) were excluded and referred elsewhere. Patients attended a brief pre-group screening appointment with the psychologist facilitating the group to provide education about the group, discuss treatment goals, and determine treatment fit. This pilot study included two groups of nine patients; however, one patient declined research participation.

The study was a longitudinal pre-post non-randomized design with a 3-month follow-up. Outcome measures were completed at weeks 1 and 8. Measures were mailed to patients with a postage paid envelope 3-months after the end of the group. Outcome measures included: (1) The Emotional Overeating Questionnaire (EOQ) (Masheb and Grilo 2006), a 9-item scale that asks respondents to rate how likely they were to overeat over the past 1 month in response to nine different emotions (e.g., anxiety, sadness) on a 7-point Likert scale (0 not likely at all; 6 extremely likely). This scale has been demonstrated as a psychometrically valid measure; (2) The Obesity Adjustment Survey (OAS) (Butler et al. 1999), a 20-item validated scale that asks respondents to rate how accurately statements describe how they have been feeling over the past 6–8 weeks in relation to their present weight and feelings, relationships, and activities, on a 5-point Likert scale (1 not at all true; 5 extremely true); (3) The Kessler Psychological Distress Scale (K10) (Andrews and Slade 2001), a 10-item validated scale that asks respondents to rate the frequency of anxiety and depression symptoms over the past 4-weeks on a 5-point Likert scale (1 all of the time; 5 none of the time; total score ranges from 10 to 50 used); (4) The Experiences in Close Relationship Scale (Short-Form; ECR-S) (Wei et al. 2007), a 12-item scale (6-items on avoidance, 6-items on anxiety), derived from the full-scale and also validated, that asks respondents to rate how much they agree with statements regarding how they generally experience relationships on a 7-point Likert scale (1 strongly disagree; 7 strongly agree); and, (5) The Outcome Questionnaire-45 (OQ-45) (Lambert et al. 1996), a widely used and validated 45-item scale that asks respondents to rate difficulties in various aspects of their lives (e.g., personal distress, relationships, and responsibilities) on a 5-point Likert scale (0 never; 4 almost always). In addition, attendance was collected and general questions on demographic information were collected at baseline. The Client Satisfaction Questionnaire (CSQ8) (Larsen et al. 1979), an 8-item measure that evaluates satisfaction along a 4-point Likert scale, was administered at week 8; patients were also asked two additional qualitative questions, including what they liked best about the group and what they would change to make it better.

## Results

Of the 17 study participants, two were male and 15 were female. Age of participants ranged from 28 to 62 years old with an average of 48 years (SD = 9.5). Most identified their maternal language as English (70.6 %) and as having graduated from college/university (70.6 %). About half were married or common-law (53 %) and about one-third (35.3 %) were working full-time, while the others were retired (23.5 %), on disability leave (17.6 %), or other (23.5 %; e.g., on social assistance).

Patients attended on average 6.7 of 8 sessions (SD = 1.8). Using the CSQ-8, satisfaction with the group was high (average of 3.37 out of 4 for the eight item total). In terms of what patients liked best about the group, respondents’ comments related to four main themes. The first, group support, spoke to the feeling of not being alone and learning from others’ experiences. They expressed valuing the learning of concrete tools for behaviour change and self-management. Patients also liked the information gained from their involvement in the group, provided through education and handouts, and the opportunity for discussion and questions. In terms of what they would change about the group, suggestions related to group timing, composition, and format. Some patients expressed wanting more frequent or lengthier sessions, others to extending overall program length. Some patients described wanting a more homogenous group (e.g., such as in terms or date since surgery or severity of psychological problems), while some others commented that they appreciated the diversity of the group. Finally, a few patients reflected on wanting more time for discussion and practicing of skills.

Data entry accuracy was verified for approximately 20 % of the data. A weighted mean was used for missing values within measures containing more than 75 % of the items. Using SPSS, a comparison of the two therapy groups involving independent sample t-tests was run to investigate for baseline differences in dependent measures; no significant differences were found. An attrition analysis involving independent sample *t*-tests was planned to investigate for possible differences in baseline dependent measures between the participants who attended 6 or more sessions and participants who attended 5 or fewer sessions; however, there were too few who attended only a few sessions and this analysis was not completed.

The effectiveness of the group on outcomes was assessed through paired samples t-tests, first comparing baseline to post-treatment periods and subsequently comparing baseline to follow-up. Due to small sample size and thus low power, no statistical correction was made and only a completer analysis was conducted. Repeated measures Cohen’s d were calculated and both effect size (0.2 small; 0.5 medium; 0.8 large) and statistical significance were considered.

Three of the six paired samples t-tests were statistically significant and had large effect sizes. Statistically significant changes were found from baseline to post-treatment on the total score for the OQ-45, the total score of the K10, and the total score of the OAS (see Table 1 for statistics). The same statistically significant changes remained when comparing participants across baseline to the 3-month follow-up time period. Specifically, participants experienced reduced level of psychological distress (K10; *t*(11) = 6.603, *p* < 0.000), reduced perceived difficulties in their lives (OQ-45; *t*(11) = 3.166, *p* < 0.009), and improved weight-related adjustment (OAS; *t*(11) = 2.780, *p* < 0.018), that remained significantly different when comparing baseline to 3 months post intervention. Furthermore, large effect sizes were found for improvements at follow-up in the OQ-45, K10, OAS, and EOQ (Cohen’s d of 0.95, 1.99, 0.83, and 0.53, respectively); small but still meaningful improvements for the ECR (Cohen’s d of 0.31 and 0.21 for anxiety and avoidance, respectively).

**Table 1:** Paired samples t-tests: comparison of scores before and after program

	N	Time 1	Time 2	t	Df	*p*	Effect size (Cohen’s d)
		Mean (SD)	Mean (SD)				
OAS total	16	55.16 (14.32)	51.31 (15.02)	2.135	15	0.050*	0.55
EOQ total	15	1.78 (1.09)	1.73 (1.31)	0.129	14	0.899	0.03
ECR-S anxiety	13	26.82 (7.09)	27.32 (6.98)	−0.321	12	0.754	0.09
ECR-S avoidance	12	24.47 (7.73)	24.08 (6.43)	0.193	11	0.850	0.06
OQ-45 total	16	79.96 (22.83)	67.53 (25.21)	4.157	15	0.001*	1.07
K10 total	16	27.28 (5.60)	22.94 (7.08)	3.024	15	0.009*	0.78

*OAS* Obesity Adjustment Survey, *EOQ* Emotional Overeating Questionnaire, *ECR*-*S* Experiences in Close Relationship Scale, *OQ-45* Outcome Questionnaire–45, *K10* Kessler Psychological Distress Scale (K10)

* Significant at the *p* value of 0.05

## Discussion and conclusions

Findings suggest that a short-term CBT psychotherapy group led to significant and meaningful benefits in psychological wellbeing for post-surgical bariatric patients. Participation in this treatment was associated with significant and meaningful improvements in patients’ level of psychological distress, perceived difficulties in their lives, and weight-related adjustment that were maintained at a three-month follow-up period. Although statistical change was not significant, there were also meaningful improvements in emotional overeating and relationship anxiety and avoidance. The intervention also appeared to be acceptable to patients in that attendance and satisfaction were good.

The limitations of this study are the same as those for other non-experimental clinical research, including absence of a control or comparison group, small sample size, and self-report nature of the findings. The non-experimental design does not permit conclusions that it was the intervention that led to the improved outcomes for patients rather than regression to the mean, placebo, or some other effect. The degree and consistency of improvements across the outcomes of interest, however, suggests that the intervention was at least in part responsible for the changes patients experienced.

The field of bariatrics represents an important emerging area of practice in mental health. The American Society for Bariatric Surgery recognizes the inclusion of mental and behavioral health experts as integral to the bariatric surgery team (LeMont et al. 2004) and clinical practice guidelines suggest that pre and post-operative assessment by mental health professionals will assist in more clearly identifying those patients who are likely to struggle adjusting to post-surgical changes (Marcus et al. 2009; Mechanick et al. 2013). This study contributes to the literature by demonstrating a short-term CBT psychotherapy group as a promising intervention for post-surgical bariatric patients. In addition to confirming the impact of this treatment in a more rigorous study design, future research exploring the cost-effectiveness of providing psychological treatment post-surgery is warranted. Research exploring a psychotherapy group that targets eating difficulties and/or interpersonal dynamics more specifically could also be worthwhile. Finally, given the emphasis placed on weight loss as a measurement of post-surgical “success”, future research would benefit from exploring the potential impact of post-operative mental health interventions on weight-related outcomes such as total weight loss, maintenance, and weight regain.
